# Serodiagnosis of Lyme borreliosis—is IgM in serum more harmful than helpful?

**DOI:** 10.1007/s10096-020-04093-2

**Published:** 2021-01-07

**Authors:** Henrik Hillerdal, Anna J. Henningsson

**Affiliations:** 1 Department of Paediatrics, Region Jönköping County, Jönköping, Sweden; 2 Department of Biomedical and Clinical Sciences, Linköping University, Linköping, Sweden; 3 Division of Clinical Microbiology, Laboratory Medicine, Region Jönköping County, Jönköping, Sweden; 4 Division of Clinical Microbiology, Linköping University Hospital, Linköping, Sweden; 5 ESCMID Study Group for Lyme Borreliosis - ESGBOR, European Society for Clinical Microbiology and Infectious Diseases, Jönköping, Sweden

**Keywords:** Clinical, Diagnostics, IgG, IgM, Lyme borreliosis, Serology

## Abstract

Interpretation of serological findings in suspected Lyme borreliosis (LB) may be challenging and IgM reactivities in serum are often unspecific (false positive). There is a risk for overdiagnosis of LB, inadequate use of antibiotics, and potential delay of proper diagnosis. In this study, we evaluated the diagnostic value of IgM analysis in serum and IgM antibody index (AI) in LB diagnosis. This was a retrospective observational study regarding *Borrelia*-specific antibodies in serum and *Borrelia*-specific AI in LB investigations being made during 2017 in Jönköping County, Sweden. Medical records of 610 patients with detectable anti-*Borrelia* antibodies in serum (IgM and/or IgG) and 15 patients with elevated *Borrelia-*specific AI were retrospectively scrutinized, and the compliance to current European recommendations was assessed. Among the 610 patients, only 30% were tested according to the European recommendations. Within this group of tests taken correctly, 50% of the LB diagnoses in patients with isolated IgM reactivity in serum were retrospectively assessed as incorrect (LB unlikely). Three pediatric patients with clinical and laboratory findings suggestive of Lyme neuroborreliosis (LNB) had elevated IgM AI alone. Serological testing without distinct clinical signs/symptoms consistent with LB contributes to most misdiagnoses. Isolated IgM positivity in serum shows limited clinical value and needs further assessment before being reported by the laboratory. Detection of IgM in combination with IgG antibodies in serum shows no clinical enhancement for correct LB diagnosis compared to isolated IgG positivity. However, *Borrelia*-specific IgM AI may be important for sensitivity in early LNB.

## Introduction

Lyme borreliosis (LB) is a tick-borne infection caused by bacteria of the *Borrelia burgdorferi* sensu lato complex. The pathology is due to an activation of the host’s innate and adaptive immune responses, [1] resulting in the production of *Borrelia*-specific antibodies. The activation of the immune system and the relative organotropism of the bacterium may lead to different but specific clinical manifestations [1, 2]. Erythema migrans (EM) which is an early localized skin infection and Lyme neuroborreliosis (LNB) constitute almost 80–90% of the cases [1, 3]. The current recommendations regarding diagnosis of LB rely upon a thorough diagnostic workup with medical history, clinical examination, and the presence of objective signs of disease, together with specific serologic findings in all manifestations but for EM, which is a clinical diagnosis since specific antibodies are detectable at this early stage in only about half of the cases [1, 4] For detection of specific antibodies, enzyme immunoassays (EIA) sometimes used with western blot for confirmation of specificity, are the mainstay for routine laboratory testing in other clinical manifestations of LB. [1, 4, 5] In patients with suspected LNB, the diagnosis requires a lumbar puncture and analysis of the cerebrospinal fluid (CSF) regarding pleocytosis and the calculation of *Borrelia-*specific CSF/serum antibody index (AI) as an indication of intrathecally produced *Borrelia-*specific antibodies [1].

The interpretation of the serologic findings (IgM and IgG) may be complicated. There are several limitations regarding the analysis of these antibodies [1–4]. For example, the prevalence of *Borrelia*-specific antibodies in the population can be over 20% in certain areas, [1, 6, 7] thus, leading to a positive test result not always being equal to active disease. Furthermore, studies have shown that IgM antibodies could lack in specificity and they can be detected due to cross-reactions with other agents, such as other spirochetes, Epstein-Barr virus, cytomegalovirus, human immunodeficiency virus, and autoantibodies [8, 9].

The clinical manifestations of LB are described in the current European case definitions in order to facilitate clinical diagnosis and enhance the highest pre-test probabilities and predictive values. [2] Despite these guidelines, in daily practice, there is a frequent overuse of laboratory testing for *Borrelia*-specific antibodies in situations where testing is not recommended, which causes a high false positive rate due to the significant seroprevalence [1]. Another contributing factor to the risk of overdiagnosis could be false positive IgM antibodies. These antibodies are relevant for detecting early infection, and according to Dessau et al [1], IgM is relevant in cases of LNB with duration < 6 weeks, Lyme carditis (LC), and Borrelial lymphocytoma (BL). The other clinical manifestations, Lyme arthritis (LA) and acrodermatitis chronica atrophicans (ACA), occur after at least 6 weeks, thus having a specific IgG response is a prerequisite. Consequently, questions have been raised regarding the clinical value of IgM testing in serum for investigating suspected LB, especially when symptoms are vague and unspecific [3]. Apprehensions are that false IgM reactivities may be misinterpreted and lead to misdiagnosis and unfounded antibiotic treatment.

The main objective of this study was to evaluate the diagnostic value of IgM analysis in serum in LB diagnosis as well as the value of IgM AI in diagnosis of LNB. The specific aims of this study were (i) to assess the proportion of serologic LB analyses ordered in adherence to current European case definitions and recommendations, (ii) to evaluate the diagnostic value of IgM in serum in LB diagnosis and of IgM AI in diagnosis of LNB, and (iii) to evaluate the accuracy of the LB diagnoses made by the clinicians after receiving the serologic results in order to assess whether a positive IgM result was helpful or harmful for the diagnosis being made.

## Materials and methods

### Study design and study subjects

**Fig. 1:**
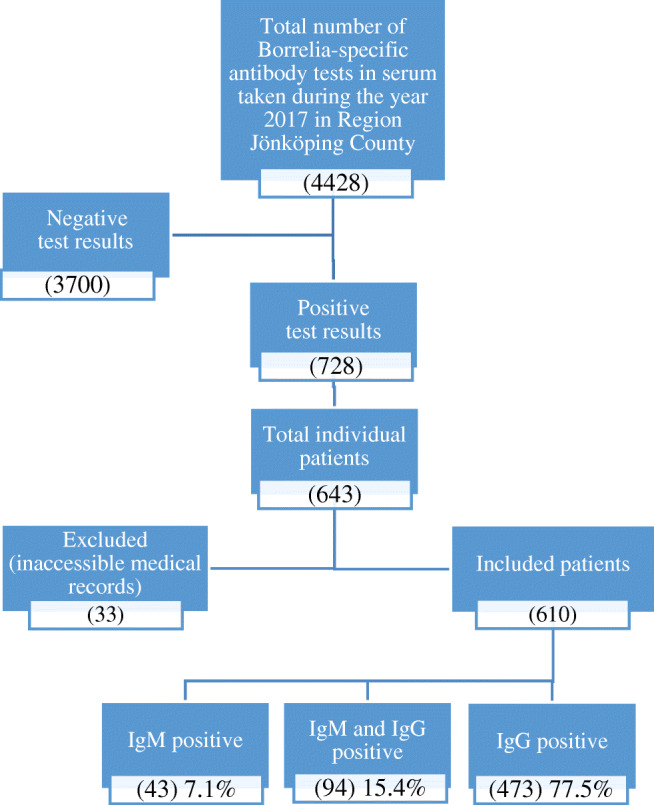
Flow chart demonstrating the inclusion process

This was a retrospective observational study regarding *Borrelia*-specific antibodies in serum and intrathecal AI in LB and LNB investigations being made during the year 2017 in Jönköping County, Sweden. There was a total number of 4428 *Borrelia*-specific antibody tests in serum analyzed during this year (Fig. 1). Of these, 3700 had negative test results, and 728 had positive results (IgM and/or IgG). The positive samples (*n* = 728) were taken from a total of 643 individual patients, of which we had to exclude 33 patients due to inaccessible medical records. Among these 610 patients included, some were tested repeatedly (*n* = 73), in which case the first positive test result during 2017 was regarded as the primary test, and any following tests taken within 6 months were only considered for evaluating potential seroconversion and not as a primary test, unless it was obvious from the medical records that the test was related to a new episode of illness with new symptoms. The patients (*n* = 610) with positive test results were divided into three separate groups depending on the seropositivity pattern (IgM positivity, IgM and IgG positivity, and IgG positivity, respectively) (Table 1).

**Table 1:** Demographic data for the 610 patients with positive serologic test results, divided in groups according to their seropositivity pattern

Seropositivity pattern	Male, *n* (%)	Female, *n* (%)	< 18 years, *n* (%)	≥ 18 years, *n* (%)	Age, median years (range)
IgM (*n* = 42)	17 (39.5)	26 (60.5)	5 (11.6)	38 (88.6)	47 (9–79)
IgM + IgG (*n* = 94)	46 (48.9)	48 (51.1)	15 (16.0)	79 (84.0)	59.8 (1–87)
IgG (*n* = 473)	265 (56.0)	208 (44.0)	39 (8.2)	434 (91.8)	66 (1–97)

Based on current European recommendations [1, 2], we defined the criteria for correct indication for serologic testing (Table 2) as being dependent on the clinical picture and medical history. We also defined the criteria for evaluation of how likely it was that the LB diagnoses made by the clinicians were correct; graded as confident, doubtful, or unlikely (Table 3). Medical records and laboratory test results for each patient were then assessed consequently according to these criteria. The specific serologic test results were not considered when assessing the indication for testing. The number of patients receiving antibiotic treatment in association with LB diagnosis was also recorded.

**Table 2:** Current European clinical case definitions of Lyme borreliosis manifestations and indications for serological testing adapted from Stanek [2] and Dessau [1]

Manifestation	Clinical case definition [2]	Laboratory evidence essential [2]	Laboratory/clinical evidence: supporting [2]	Detection of antibodies to *Borrelia burgdorferi* s.l. [1]
Erythema migrans	Expanding red or bluish-red rash (≥ 5 cm in diameter)^a^ with or without central clearing. Advancing edge typically distinct, often intensely colored, not markedly elevated.	None	Detection of *B. burgdorferi* s.l. by culture and/or PCR from skin biopsy.	Not recommended
Borrelial lymphocytoma	Painless bluish-red nodule or plaque, usually on ear lobe, ear helix, nipple, or scrotum; more frequent in children (especially on ear) than in adults.	Seroconversion or positive serology^b^ Histology in unclear cases	Histology. Detection of *B. burgdorferi* s.l. by culture and/or PCR from skin biopsy. Recent or concomitant EM.	Serum IgG and/or IgM
Acrodermatitis chronica atrophicans	Long-standing red or bluish-red lesions, usually on the extensor surfaces of extremities. Initial doughy swelling. Lesions eventually become atrophic. Possible skin induration and fibroid nodules over bony prominences.	High level of specific serum IgG antibodies	Histology. Detection of *B. burgdorferi* s.l. by culture and/or PCR from skin biopsy.	Serum IgG
Lyme neuroborreliosis	In adults, mainly meningo-radiculitis, meningitis; rarely encephalitis, myelitis; very rarely cerebral vasculitis. In children mainly meningitis and facial palsy.	Pleocytosis and demonstration of intrathecal-specific antibody synthesis^c^	Detection of B. burgdorferi s.l. by culture and/or PCR from CSF. Intrathecal synthesis of total IgM, and/or IgG and/or IgA. Specific serum antibodies. Recent or concomitant EM.	Specific CSF/serum antibody index
Lyme arthritis	Recurrent attacks or persisting objective joint swelling in one or a few large joints. Alternative explanations must be excluded.	Specific serum IgG antibodies, usually in high concentrations	Synovial fluid analysis. Detection of B. burgdorferi s.l. by PCR and/or culture from synovial fluid and/or tissue.	Serum IgG
Lyme carditis	Acute onset of atrio-ventricular (I–III) conduction disturbances, rhythm disturbances, sometimes myocarditis or pancarditis. Alternative explanations must be excluded	Specific serum antibodies	Detection of B. burgdorferi s.l. by culture and/or PCR from endomyocardial biopsy. Recent or concomitant erythema migrans and/or neurologic disorders.	Serum IgG and/or IgM
Ocular manifestations	Conjunctivitis, uveitis, papillitis, episcleritis, keratitis.	Specific serum antibodies	Recent or concomitant Lyme borreliosis manifestations. Detection of B. burgdorferi s.l. by culture and/or PCR from ocular fluid.	Serum IgG

^a^If < 5 cm in diameter, a history of tick bite, a delay in appearance (after the tick bite) of at least 2 days, and an expanding rash at the site of the tick bite is required

^b^As a rule, initial and follow-up samples have to be tested in parallel in order to avoid changes by inter-assay variation

^c^In early cases, intrathecally produced specific antibodies may still be absent

*EM*, erythema migrans; *B. burgdorferi s.l.*, Borrelia burgdorferi sensu lato; *CSF*, cerebrospinal fluid; *PCR*, polymerase chain reaction

**Table 3:** Criteria for estimating the likelihood and accuracy of the potential diagnosis of Lyme borreliosis (LB) made by the clinician [1, 2, 4]

LB diagnosis	Objective clinical signs consistent with LB (Table 1)	Serology supporting the suspected manifestation (Table 1)	Seroconversion or significant elevation of antibodies^a^
Confident	Yes	Yes	Yes
Doubtful	Yes	No	No
Doubtful	No	Yes	No
Unlikely	No	No	No

^a^These parameters were assessed when available. Seroconversion, from negative to positive (IgM or IgG). Significant elevation of antibodies, a 2-fold increase or more of the antibody level (optical density)

In addition, we looked into all analyses of LNB performed on paired serum and CSF samples during 2017 (*n* = 579). We selected all patients with positive *Borrelia*-specific CSF/serum AI (*n* = 15) and divided them into similar groups according to their positive AI (IgM AI and/or IgG AI). These groups were similarly assessed by their medical records regarding symptoms, present CSF pleocytosis, LNB diagnosis given, antibiotic treatment, and if they had *Borrelia-*specific antibodies detected in serum as well. Pleocytosis was defined as total white blood cell count > 5 × 10^6^/L in CSF.

### Serologic assays

Patient samples had been analyzed for *Borrelia*-specific antibodies previously and in accordance with local routines at the Laboratory of Clinical Microbiology, Region Jönköping County, Sweden. The assays used for serum samples were Enzygnost Borrelia Lyme IgM and Enzygnost Borrelia Lyme link VlsE/IgG (Siemens/DADE Behring, Marburg, Germany). The cut-off levels used were 10 U/mL for IgG and 2 U/mL for IgM. The IgM cut-off had after evaluation been adapted to local seroprevalence conditions (the cut-off suggested by the manufacturer is 1 U/mL). Seroconversion was defined as *Borrelia*-specific antibodies being undetectable in the first sample but positive in a second sample (if available). Significant elevation of antibodies was in this study defined as a 2-fold increase or more of the antibody level (optical density (OD)). The authors are aware that an increase of the OD above the linear range of the assay is not possible to assess.

For paired serum and CSF samples, the *Borrelia*-specific AI was determined by the IDEIA Lyme neuroborreliosis kit (IgM and IgG) (Oxoid, Hampshire, UK), which is a flagella antigen-based enzyme linked immunosorbent assay. A *Borrelia*-specific AI > 0.3 was considered as a positive result.

## Results

### Assessment of patients with *Borrelia*-specific antibodies detected in serum samples

Among the patients with positive *Borrelia* serology (IgM and/or IgG), 183/610 (30 %) were tested according to the European clinical recommendations (Table 4). Of these patients, 96/183 (52.5 %) received a LB diagnosis, and of them 90/96 (93.8 %) were considered either confident 66/96 (68.8 %) or doubtful 24/96 (25 %), whereas only 6/96 (6.3 %) of the diagnoses being made were assessed as unlikely. The distribution of different LB manifestations among the diagnoses made by clinicians was devided as seen in Table 5.

**Table 4:** Classification of diagnoses being made in each of the patient groups based on serological results, stratified according to whether serological testing was performed in agreement with recommendations or not

	Tests following guidelines 30% (183 patients)		Tests not following guidelines 70% (427 patients)			
			EM diagnoses included (*n* = 427)		EM diagnoses excluded (*n* = 353)	
	^a^	^b^ (%)	^a^	^b^ (%)	^a^	^b^ (%)
Total	96/183	52.5	134/427	31.4	72/353	20.4
Confident	66/96	68.8	47/134	35.1	0/72	0.0
Doubtful	24/96	25.0	51/134	38.1	37/72	51.4
Unlikely	6/96	6.3	36/134	26.9	35/72	48.6
IgM	4/11	36.4	18/32	56.3	9/23	39.1
Confident	1/4	25.0	9/18	50.0	0/9	0.0
Doubtful	1/4	25.0	5/18	27.8	5/9	55.6
Unlikely	2/4	50.0	4/18	22.2	4/9	44.4
IgM + IgG	30/32	93.8	50/62	80.6	22/34	64.7
Confident	22/30	73.3	21/50	42.0	0/22	0.0
Doubtful	7/30	23.3	22/50	44.0	15/22	68.2
Unlikely	1/30	3.3	7/50	14.0	7/22	31.8
IgG	62/140	44.3	66/333	19.8	41/296	13.9
Confident	43/62	69.4	17/66	25.8	0/41	0.0
Doubtful	16/62	25.8	24/66	36.4	17/41	41.5
Unlikely	3/62	4.8	25/66	37.9	24/41	58.5

^a^Number of LB diagnoses/number of positive test results

^b^Percentage of positive test results ending up in a diagnosis of LB

**Table 5:** The distribution of different LB manifestations suspected by the clinicians, here being retrospectively assessed as confident, doubtful, or unlikely in cases where serological testing was performed according to recommendations compared to tests taken in contradiction to recommendations

	Tests following guidelines (*n* = 183)				Tests not following guidelines (*n* = 427)			
	Confident	Doubtful	Unlikely	No. diagnosis given	Confident	Doubtful	Unlikely	No. diagnosis given
EM	0	0	0	0	46	14	1	12
LNB	19	6	1	70	0	21	6	53
LA	10	4	0	7	0	3	13	43
BL	7	4	1	2	0	1	0	0
ACA	30	9	3	5	0	0	0	0
LC	0	0	1	3	0	0	0	0
Unspec	0	1	0	0	1	12	16	185

*EM*, erythema migrans; *LNB*, Lyme neuroborreliosis; *LA*, Lyme arthritis; *BL*, borrelial lymphocytoma; *ACA*, acrodermatitis chronica atrophicans; *LC*, Lyme carditis; *Unspec*, unspecified suspected diagnosis

**Fig. 2:**
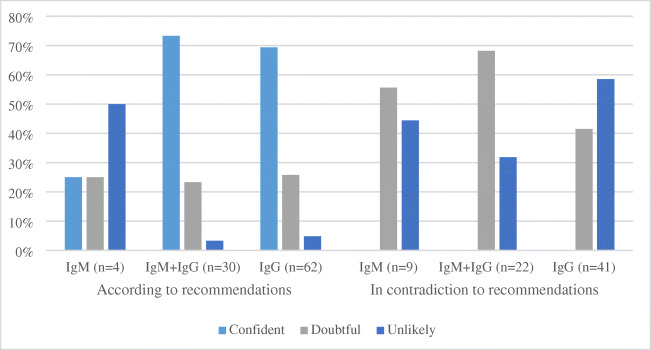
The proportions of LB diagnoses being assessed as confident, doubtful, or unlikely in cases where serological testing was performed according to recommendations compared to tests taken in contradiction to recommendations (EM diagnoses excluded)

In the samples tested according to current recommendations, the groups positive for either isolated IgG or both IgM and IgG antibodies showed a similar pattern with high number of diagnoses assessed as being confident or doubtful (Fig. 2). The same patterns were noticed when children and adults were analyzed separately (data not shown). In contrast, isolated detection of IgM (without concomitant IgG) was only helpful in 50% of the diagnoses assessed as being confident or doubtful (Fig. 2). Thus, 50% of the LB diagnoses in patients with isolated IgM reactivity in serum were assessed as incorrect (LB unlikely) in the group of patients where testing had been performed in accordance with current European recommendations. A total number of 40 repeated tests were taken within 6 months from the primary test, and in this group, 9 patients fulfilled the criteria for seroconversion or significant elevation of antibodies (8 patients with LNB and 1 patient with LA).

We found that 427/610 (70 %) patients were tested for *Borrelia*-specific antibodies in contravention of current clinical recommendations. Among them, 74/427 (17.3%) had EM and should not have been serologically tested. After excluding patients with EM, 72/353 (20.4%) of the remaining patients received a LB diagnosis (Table 4), and of them 37/72 (51.4 %) were considered doubtful, and 35/72 (48.6 %) of the diagnoses being made were assessed as unlikely. None of the diagnoses were considered confident (Fig. 2). Forty-four repeated tests were taken within 6 months after the primary test, and of them, only one showed seroconversion. This patient presented with EM.

Almost every patient who received a LB diagnosis was treated with antibiotics; 95/96 (99.0 %) among the patients where tests were taken according to recommendations and 71/72 (98.6 %) in the group of patients where tests were taken outside the current recommendations.

### Assessment of patients with *Borrelia*-specific antibodies in paired serum and CSF samples

Among the CSF/serum analyses, 15/579 (2.6%) patients had an elevated AI (IgG AI *n* = 6, IgM AI *n* = 3, IgG and IgM AI *n* = 6) and CSF pleocytosis were present in all but in 3 patients, all within the IgG AI group (Table 6). In the group of IgM AI, 2/3 patients had no detectable levels of *Borrelia*-specific antibodies in serum. Confident diagnosis and treatment were given to patients with pleocytosis in CSF. Regarding the three patients with absence of CSF pleocytosis, 2/3 were given a doubtful diagnosis and treatment, and 1/3 was considered to be a previous infection and no treatment was given.

**Table 6:** Showing all patients with a positive *Borrelia*-specific CSF/serum antibody index (*n* = 15) and their respective characteristics

Positive *Borrelia*-specific CSF/serum AI	Year of birth	Positive *Borrelia*-specific antibodies in serum	Pleocytosis	Diagnosis given	Estimation of likelihood and accuracy of given diagnosis	Treatment with antibiotics
IgG	1923	IgG	No	Yes	Doubtful	Yes
IgG	2014	Negative	No	Yes	Doubtful	Yes
IgG	1959	IgM and IgG	Yes	?^a^	?^a^	?^a^
IgG	1965	IgG	No	No	-	No
IgG	1967	IgM and IgG	Yes	Yes	Confident	Yes
IgG	1938	IgG	Yes	Yes	Confident	Yes
IgM	2009	Negative	Yes	Yes	Confident	Yes
IgM	2009	Negative	Yes	Yes	Confident	Yes
IgM	2013	IgM and IgG	Yes	Yes	Confident	Yes
IgM and IgG	1974	IgM and IgG	Yes	Yes	Confident	Yes
IgM and IgG	2009	IgG	Yes	Yes	Confident	Yes
IgM and IgG	1995	IgM and IgG	Yes	Yes	Confident	Yes
IgM and IgG	2011	IgG	Yes	Yes	Confident	Yes
IgM and IgG	1936	IgG	Yes	Yes	Confident	Yes
IgM and IgG	2010	IgM and IgG	Yes	Yes	Confident	Yes

^a^Not possible to obtain accurate information due to inaccessible medical records

## Discussion

In this study, we evaluated the clinical impact of IgM reactivities in *Borrelia* testing of serum and CSF, and whether the results actually enhance the clinicians’ way to a proper diagnosis or rather misguide them. By looking at all positive *Borrelia-*specific antibody tests in serum in Jönköping County during the year 2017, we found that merely 30% of the tests were taken according to current guidelines. The proportion of borrelia tests taken in accordance with the recommendations is presumably even lower in the group with negative test results (*n* = 3700), but this has not been investigated here. This finding is similar to what has been shown in other studies where as much as up to 82% of tests had been taken in contradiction with current guidelines [10, 11] Most certainly, this fact emphasizes that serological testing without distinct clinical signs/symptoms consistent with LB contributes to most misdiagnoses.

When looking at the results of the tests taken according to recommendations, there is no clinical enhancement for dual IgM and IgG positivity compared to isolated IgG positivity to be seen when it comes to establishing a correct diagnosis (Fig. 2). Hence, no added value of IgM detection in serum could be seen in this group.

Furthermore, the proportion of patients receiving a LB diagnosis was higher in the group with both IgM and IgG positivity compared to the group with isolated IgG positivity, and as seen during our review of the patients’ medical records, clinicians tend to believe that a present IgM response is required for an active infection, whereas the lack of IgM positivity rather speaks against an active infection. In several cases with IgG positivity alone, the physicians had decided not to treat the patient with antibiotics even though the LB diagnosis seemed quite obvious with specific symptoms and correct serologic findings. As an example, ACA was ruled out in a patient because the lack of IgM antibodies even though a long-standing skin rash and high *Borrelia*-specific IgG levels were present.

Isolated IgM positivity in serum is quite rare in the group of patients tested in accordance with the recommendations. Isolated IgM showed very limited clinical value and needs further assessment in order to offer any guidance at all. The analyzing laboratory should either have a routine of confirming specificity with an immunoblot, or instead recommend a follow-up test 4– weeks later. In suspected LNB cases, a lumbar puncture should be performed and the presence of intrathecally produced *Borrelia*-specific antibodies should be investigated.

When analyzing the borrelia tests taken without proper indication, we noticed the same pattern regarding the presence of IgM positivity and its effect on whether a LB diagnosis was made or not. Clinicians connect a positive IgM with an active infection and are more inclined to interpret it as an actual and on-going infection, regardless of the lack of specific symptoms. The result is that patients acquire a very doubtful diagnosis and receive treatment with antibiotics on incorrect grounds. Isolated IgM reactivities in the group of patients tested outside current recommendations, EM patients excluded, were more frequently assessed as unspecific in this study.

In this perspective, IgM reactivities, as well as lack of them, seem to be more harmful than helpful in LB diagnosis, causing both over- and underdiagnosis, overuse of antibiotics and delay of proper diagnosis and treatment. For the patients, delay of correct diagnosis may be associated with prolonged suffering and anxiety, and for the healthcare system, with increased costs.

In our patients with paired serum and CSF analyses, we noticed that among 15 patients with positive *Borrelia*-specific CSF/serum AI, 3/11 confident diagnoses of LNB would have been missed if IgM AI analyses had not been performed. All three patients were children, two with symptoms of meningitis and one with a one sided facial palsy, and all had elevated IgM AI only. Thus, determination of *Borrelia-*specific IgM AI increased the diagnostic sensitivity for the IDEIA Lyme neuroborreliosis assay. Furthermore, two of these three children had no detectable levels of *Borrelia*-specific antibodies in serum, which underscores the necessity of performing CSF analysis in suspected LNB cases.

Our findings suggest that IgM testing in serum is potentially more harmful than helpful in LB diagnosis. However, the main problem seems to be the large amount of tests taken outside the current recommendations, i.e., with very low pre-test-probability, and our suspicion is that many clinicians lack the proper knowledge regarding how to use and interpret serologic findings in LB diagnosis, and have very high faith in especially IgM positivity. Based on our assessments in this study, our conclusion is that IgM testing in serum causes more unreliable diagnoses and mistreatments, and should therefore be excluded in future testing, except when the analysis is paired with a simultaneous CSF analysis. Perhaps, another possibility could be that the laboratory would demand explicit and distinct clinical information on the referral and only perform IgM testing in cases where it actually could be useful, such as early LNB, LC, and BL as suggested by Dessau et al [1]. At least, isolated IgM reactivities in serum samples should not be reported uncritically by the laboratory without further confirmation, either with immunoblot or another EIA with different antigen composition.

## Conclusions

Based on our findings, we conclude that IgM analysis in serum is of limited diagnostic value for LB diagnosis since no increase of sensitivity is gained compared to IgG testing alone. In addition, specificity issues may lead to misinterpretation and overdiagnosis of LB. In contrast, analysis of *Borrelia*-specific AI may be important for sensitivity in early LNB. However, the extensive testing for *Borrelia*-specific antibodies in contravention of current recommendations appears to be a major factor complicating interpretation of serological results and contributing to misdiagnosis and unfounded use of antibiotics.

## Data Availability

Original data is available from the corresponding author upon request.

## References

[1] Dessau RB, van Dam AP, Fingerle V, Gray J, Hovius JW, Hunfeld KP, Jaulhac B, Kahl O, Kristoferitsch W, Lindgren PE, Markowicz M, Mavin S, Ornstein K, Rupprecht T, Stanek G, Strle F (2018). To test or not to test? Laboratory support for the diagnosis of Lyme borreliosis: a position paper of ESGBOR, the ESCMID study group for Lyme borreliosis. Clin Microbiol Infect.

[2] Stanek G, Fingerle V, Hunfeld KP, Jaulhac B, Kaiser R, Krause A, Kristoferitsch W, O'Connell S, Ornstein K, Strle F, Gray J (2011). Lyme borreliosis: clinical case definitions for diagnosis and management in Europe. Clin Microbiol Infect.

[3] Bremell D, Jacobsson G (2018) Minska antalet borreliaserologier. Läkartidningen; 115:E46E. https://lakartidningen.se/opinion/debatt/2018/04/minska-antalet-borreliaserologier/29634074

[4] Smittskyddsinstitutet (2013) Laboratoriediagnostik av borreliainfektion. Smittskyddsinstitutet. https://www.folkhalsomyndigheten.se/publicerat-material/publikationsarkiv/l/laboratoriediagnostik-av-borreliainfektion/. Accessed 05 Oct 2018

[5] Swedish Medical Products Agency (2009) Läkemedelsbehandling av borreliainfektion. Information från Läkemedelsverket, 2009;20(4). https://www.lakemedelsverket.se/48e68d/globalassets/dokument/publikationer/information-fran-lakemedelsverket/information-fran-lakemedelsverket-nr-4-2009.pdf. Accessed 24 Oct 2018

[6] Mygland A, Skarpaas T, Ljostad U (2006). Chronic polyneuropathy and Lyme disease. Eur J Neurol.

[7] Tjernberg I, Kruger G, Eliasson I (2007). C6 peptide ELISA test in the serodiagnosis of Lyme borreliosis in Sweden. Eur J Clin Microbiol Infect Dis.

[8] Ang CW, Notermans DW, Hommes M, Simoons-Smit AM, Herremans T (2011). Large differences between test strategies for the detection of anti-Borrelia antibodies are revealed by comparing eight ELISAs and five immunoblots. Eur J Clin Microbiol Infect Dis.

[9] Busson L, Reynders M, Van den Wijngaert S, Dahma H, Decolvenaer M, Vasseur L, Vandenberg O (2012). Evaluation of commercial screening tests and blot assays for the diagnosis of Lyme borreliosis. Diagn Microbiol Infect Dis.

[10] Coumou J, Hovius JW, van Dam AP (2014). Borrelia burgdorferi sensu lato serology in the Netherlands: guidelines versus daily practice. Eur J Clin Microbiol Infect Dis.

[11] Dessau RB, Bangsborg JM, Ejlertsen T, Skarphedinsson S, Schønheyder HC (2010). Utilization of serology for the diagnosis of suspected Lyme borreliosis in Denmark: survey of patients seen in general practice. BMC Infect Dis.

